# Special Issue: Insects, Nematodes, and Their Symbiotic Bacteria

**DOI:** 10.3390/insects11090577

**Published:** 2020-08-28

**Authors:** Ulrich Theopold, Alexis Dziedziech, Pavel Hyrsl

**Affiliations:** 1 Department of Molecular Biosciences, Wenner-Gren Institute, Stockholm University, Svante Arrhenius Väg 20C, 10691 Stockholm, Sweden; alexis.dziedziech@su.se; 2 Department of Experimental Biology, Faculty of Science, Masaryk University, Kamenice 753/5, 625 00 Brno, Czech Republic; hyrsl@sci.muni.cz

**Keywords:** insect immunity, entomopathogenic nematodes, immunity, symbiotic bacteria, *Heterorhabditis*, *Steinernema*, *Photorhabdus*, *Xenorhabdus*

## Abstract

This special issue contains articles that add to the ever-expanding toolbox of insect pathogenic nematodes (entomopathogenic nematodes; EPNs) as well articles that provide new insights into the mutualistic interaction between EPNs and their hosts. The study of natural infection models such as EPNs allows detailed insight into micro- and macro-evolutionary dynamics of innate immune reactions, including known but also emerging branches of innate immunity. Additional new insights into the kinetics of EPN infections are gained by increased spatiotemporal resolution of advanced transcriptome studies and live imaging.

## 1. Introduction

Parasite–host interactions are characterized by a constant race between both parts involved. In the case of entomopathogenic nematodes (EPNs), this is further complicated by the involvement of a third partner, namely their endosymbiotic bacteria, which contribute to the nematodes’ success. This makes EPNs a highly effective but also versatile tool to control insect pest species, with EPNs often used as part of integrated pest management (IPM). On the other hand, an increased understanding of their mutual tripartite interactions holds the promise to offer new insight into the workings of insect immunity and the virulence strategies that are deployed by nematodes and their bacteria. The selection of articles in this special issue aims to both identify tools that improve the understanding and use of EPNs as biological control agents as well as articles that describe insect immune responses that are protective in some host–pathogen combinations but are rendered ineffective by some EPNs.

## 2. Routes into Novel Applications and Eco-Evolutionary Studies

One of the advantages of EPNs is their widespread distribution, which allows the identification of new species or populations from different environments and their characterization. This provides an increasing dataset for immune-ecological and evolutionary studies. One such example is the isolation and initial characterization by Özdemir and co-workers of *Steinernema litorale* and its endosymbiotic bacterium from Ankara, Turkey [1], which provides an additional tool for IPM and useful data for microevolutionary studies. Similarly, Modic et al. [2] compare the efficacy and persistence of EPNs (*Heterorhabditis bacteriophora*) against western corn rootworm (*Diabrotica virgifera virgifera*) larvae with the use of insecticides under divergent climatic conditions, soil pest densities, and modes of application. When it comes to the question of how insect hosts respond, Darsouei et al. [3] find that outer membrane proteins (OMPs) from two endosymbiotic bacteria (*Xenorhabdus nematophila* and *Photorhabdus luminescens*) induce partially overlapping but distinct responses in their insect hosts. This may qualify OMPs as genuine virulence factors that participate in host–pathogen competition similar to OMPs from other pathogenic bacteria. 

## 3. Branches of Insect Immunity That Are Targeted by EPNs

An often too little appreciated role is the contribution of eicosanoids towards insect immunity. In pioneering work, Kim and co-workers showed that eicosanoid signaling is targeted by EPNs and their bacteria (summarized in [4]). This has now been confirmed for *Steinernema feltiae* and—after using antibiotic treatment—partly attributed to their bacteria, although nematodes seem to be contributing too [5]. In addition to a reduction in cellular immunity, the activation of prophenoloxidase is used in this article as a readout for immune competence. Activation of prophenoloxidase is also shown to be targeted by secreted products that are released by EPNs [6]. Elias et al. go on to further characterize the secreted factors at the molecular level. In two review articles, the recent work of Dziedziech et al. [7] and Eleftherianos et al. [8] is summarized for two branches of insect immunity, namely the coagulation of hemolymph and the role of thioester-containing proteins in the response against EPNs. In the work of both groups, the fruit fly *Drosophila melanogaster* features prominently, opening the route into molecular genetic analysis of EPN–host interactions. In a second article, Dziedziech et al. [9] use the fruit fly to get a more detailed insight into the early stages of EPN infection using high-resolution microscopy and tracking of infected larvae. Finally, one article on the use of an entomopathogenic fungus (*Metarhizium anisopliae*) is included to remind us of a similar route of infection via epithelial surfaces used by both fungi and EPNs [10]. Jiang et al. also bring us back to the use of both types of pathogens as biological control agents—much needed tools to control major outbreaks such as the locust outbreak experienced in Eastern Africa at the time of writing. 

## 4. Conclusions

As evidenced from the collection of articles in this special issue, studies on EPNs provide an almost unique opportunity to combine insights into their practical use with the characterization of the virulence factors and their targets at the molecular level. For some EPNs, this is facilitated by the availability of genome data for all partners involved in the tripartite interaction between nematodes, their bacteria, and the insect hosts. Further, the study of EPNs bridge more functional taxonomical studies with high-throughput molecular methods that are increasingly available. Thus, natural infections such as with EPNs (and naturally occurring fungi) provide new perspectives on the dynamics of host–parasite interactions, expand our view of the insects’ toolbox of immunity, and demonstrate the complexities of co-dependent evolution. 
